# Exploring Genetic Influences on Equine Meat Quality: A Bioinformatics Approach

**DOI:** 10.3390/foods14030533

**Published:** 2025-02-06

**Authors:** Martin Šimon, Sanja Bogićević, Ana Kaić, Barbara Luštrek, Klemen Potočnik

**Affiliations:** 1 Department of Animal Science, Biotechnical Faculty, University of Ljubljana, Groblje 3, 1230 Domžale, Slovenia; martin.simon@bf.uni-lj.si (M.Š.); sanja.bogicevic@bf.uni-lj.si (S.B.); barbara.lustrek@bf.uni-lj.si (B.L.); klemen.potocnik@bf.uni-lj.si (K.P.); 2 Department of Animal Science and Technology, Faculty of Agriculture, University of Zagreb, Svetošimunska 25, 10000 Zagreb, Croatia

**Keywords:** bioinformatics, equine genomics, horsemeat production, horsemeat quality, selective breeding, SNP markers

## Abstract

Horsemeat, known for its high nutritional value and lower environmental impact compared to beef, faces cultural and ethical challenges. Despite its potential, genetic research on horsemeat quality remains limited and no Quantitative Trait Loci (QTLs) have been identified. The aim of this study was to identify and prioritize Single Nucleotide Polymorphism (SNP) markers on the GeneSeek^®^ GenomicProfiler™ Equine chip for traits related to meat quality. Genes associated with meat quality were identified through a PubMEd search. These were analyzed for SNPs with potential regulatory or functional effects based on Genomic Evolutionary Rate Profiling (GERP) scores, constrained element locations, orthologous regulatory regions in mice and humans, and effects on polyadenylation, miRNA, and transcription factor binding. Further prioritization focused on genes whose orthologs are within QTLs for meat quality traits in other species. Including SNPs in linkage disequilibrium with chip markers from the Animal-SNPAtlas, we identified 27 SNP markers associated with 19 genes. Notable candidates include *ALDOA*, *CS*, *GOT1*, *PLIN1*, *PYGM*, and *SDHB*, linked to metabolic pathways, and *MYL11*, *MYOM1*, *PDLIM5*, *RYR3*, and *TNNT3*, associated with muscle structure and development. This research provides genetic insights to improve horsemeat quality and help breeders and smallholder farmers. Integrating these results with larger datasets can improve breeding value predictions and support effective breeding programs.

## 1. Introduction

Meat has long played a fundamental role in human nutrition, providing essential nutrients necessary for growth, development, and health. As global populations grow and dietary habits shift, the demand for meat continues to rise. In 2023, global meat production surpassed 370 million tons [1], and projections estimate this figure will reach between 460 and 570 million tons by 2050 [2]. However, this increase presents significant environmental challenges, highlighting the urgent need for sustainable protein alternatives to mitigate the strain on resources and reduce greenhouse gas emissions [3].

Due to the relatively low production of horsemeat, it cannot serve as a universal alternative to all types of red meat. However, given the increasing demand for alternative meats compared to conventional options, horsemeat could play an important role as a substitute for red meat, especially beef, and could be promoted as a ‘dietary’ meat option [4]. Although it represents only a small proportion of global meat production, horsemeat offers several advantages. Its production has a lower environmental impact compared to that of ruminants, mainly because horses emit significantly less methane due to their unique digestive physiology [3,5]. This makes horsemeat a more sustainable protein source in the context of climate change and resource conservation.

From a nutritional point of view, horsemeat is extremely competitive. It is low in fat (2.9%), high in protein (22.5%) and rich in unsaturated fatty acids (over 55%). It also contains high levels of bioavailable iron, which contributes to its potential positive effects on human health [4,6,7]. These properties make it an attractive option for health-conscious consumers looking for nutrient-rich and lower-calorie protein sources.

Despite its benefits, the consumption of horsemeat varies considerably around the world. In 2023, the global average per capita supply worldwide was around 0.10 kg. However, some countries such as Mongolia (11.97 kg), Kazakhstan (8.38 kg), and Kyrgyzstan (4.40 kg) reported much higher values [1]. In Europe, on the other hand, consumption is moderate in countries such as Belgium, Italy, and Switzerland, often due to the use of horsemeat in traditional products such as dried meat and sausages [3,8]. The cultural acceptance and market potential of horsemeat depends heavily on its perceived quality, nutritional profile, and culinary applications.

The quality of horsemeat is determined by various factors, including tenderness, color, and muscle fiber composition. Tenderness, one of the most important attributes for consumer acceptance, which is often measured by shear force, can vary greatly in horsemeat, ranging from ‘very tender’ to ‘tough’. Studies confirm that consumers can recognize these differences and are willing to pay a premium for more tender meat [9]. The tenderness of horsemeat is also influenced by age, with younger animals producing more tender meat due to their lower collagen and connective tissue content [6,10]. Additionally, the composition of muscle fibers also influences meat quality. Oxidative fibers (type I and type IIa) are associated with greater tenderness, improved water holding capacity and a bright red color, making them desirable for meat production. In contrast, glycolytic fibers (type IIb) are associated with tougher meat with higher shear force and a paler appearance. The balance between these muscle fiber types is therefore crucial for optimizing meat quality [11,12].

Advances in transcriptomics and proteomics have provided deeper insights into the molecular mechanisms underlying horsemeat quality. Transcriptomic studies have identified key genes such as *ACTN3*, *MYOZ2*, and *SLN* that regulate the transition between muscle fiber types and influence traits such as tenderness and color [13,14]. Similarly, proteomic analyses have highlighted the role of specific proteins in energy metabolism and structural integrity during meat aging, providing valuable targets for improving quality through breeding and processing techniques [15].

Despite these advances, research in equine genetics and meat production remains limited. This knowledge gap is evident in databases such as Animal QTLdb, which lack comprehensive data on equine-specific production traits. Addressing this dearth is critical to using genetic selection to improve the quality and sustainability of horsemeat production.

The main objective of this study is to use a commercial SNP microarray (GeneSeek^®^ Genomic Profile) to identify and prioritize single nucleotide polymorphism (SNP) markers relevant to horsemeat quality. By focusing on traits such as tenderness, color, and muscle structure, this research will provide a basis for genetic selection strategies. The results will enable breeders and researchers to make data-driven decisions to improve the production of high-quality horsemeat while supporting sustainable animal husbandry practices. In addition, the findings from this study provide a foundation for the application of genomic tools, such as genomic Best Linear Unbiased Prediction (gBLUP) and genome-wide association studies (GWAS), to enhance our understanding of the genetic factors influencing horsemeat quality.

## 2. Materials and Methods

### 2.1. Literature Overview

We conducted a systematic search in the PubMed database on 25 October 2024, to collect research results related to proteomic and transcriptomic data on meat quality and muscle/fiber types in horses and donkeys. The search query used was as follows: (horse*[Title/Abstract] OR equus[Title/Abstract] OR equidea[Title/Abstract] OR donkey[Title/Abstract]) AND (horsemeat[Title/Abstract] OR “horse meat”[Title/Abstract] OR muscle[Title/Abstract] OR meat[Title/Abstract]) AND (proteo*[Title/Abstract] OR transcrip*[Title/Abstract] OR express*[Title/Abstract] OR differential[Title/Abstract] OR abundance[Title/Abstract])

This search yielded 673 results, which were manually reviewed based on title and abstract relevance. We excluded studies that did not focus on proteomic or transcriptomic data relevant to meat quality and muscle characteristics in horses and donkeys. Many studies focused on differentiating horsemeat from other meats rather than analyzing its molecular determinants. After applying these criteria, we identified 10 key studies for in-depth analysis, which were incorporated into our study.

### 2.2. SNP Identification

#### 2.2.1. SNP Markers on the GGP Equine

We first obtained stable IDs for the identified genes and proteins specifically in horses using the Ensembl BioMart (Release 113, [16]), National Center for Biotechnology Information (Release 263, [17]) and UniProt (Release 2024_06, [18]) databases. While the analysis focused on horses, orthologs from donkeys were also included to provide a broader genomic context. This comprehensive approach ensured that the relevant genes were included for a robust analysis of meat quality traits.

The Ensembl BioMart tool (Release 113, [16]) was then used to obtain the genomic coordinates of candidate genes in horses based on the EquCab3.0 reference genome. To identify potential regulatory variants, an interval of 5000 bp was extended upstream and downstream of the coordinates of each gene. This extended range allows the inclusion of variants located in potential regulatory regions [19,20,21]. Markers within these regions were extracted from the GeneSeek^®^ Genomic Profiler™ Equine, which contains over 70,000 SNPs. These markers were then analyzed using the bioinformatics tools described below.

#### 2.2.2. SNPs in Linkage Disequilibrium

To include relevant SNP markers that might otherwise be neglected, we identified SNPs from the Animal-SNPAtlas database (accessed 20 November 2024, [22]) that are in linkage disequilibrium (LD) with markers on the SNP chip. Even if the marker itself does not meet the prioritization criteria, the SNP linked to it in LD could meet the requirements and have functional significance. Only SNPs with an LD threshold of R^2^ > 0.95 were considered, based on data from 87 horses from 25 breeds. By including LD information, this approach ensures that both meaningful variants from whole genome sequencing data and the associated markers on the chip are considered.

#### 2.2.3. Predicted Variant Consequences of SNPs

The Ensembl Variant Effect Predictor (VEP) (Release 113, [23]) was used to predict the variant consequences for both the selected markers and the SNPs in linkage disequilibrium.

#### 2.2.4. SNPs Within Evolutionarily Constrained Elements

To identify SNPs (both markers and SNPs in LD) located within constrained elements, we used the file gerp_constrained_elements.equus_caballus.bb downloaded from the Ensembl FTP site (Release 113, [16]). This file, which annotates conserved regions derived from GERP scores, was converted to a BED file using the bigBedToBed tool from the UCSC Genome Browser Utilities [24]. The genomic coordinates of the SNPs were then compared to the constrained regions using RStudio (version 2024.04.2, Posit Software, PBC, Boston, United States) to identify overlaps. Using this approach, we were able to locate SNPs in evolutionarily conserved regions, highlighting their potential functional significance.

#### 2.2.5. SNPs with High Evolutionary Conservation Using GERP Scores

To prioritize SNPs (both markers and SNPs in LD) with high evolutionary conservation, we analyzed their GERP scores, defined as the decrease in the number of substitutions observed in a multispecies sequence alignment compared to the neutral expectation [25]. Positive GERP scores of 2 or higher are generally considered significant and reflect increased conservation and biological significance [26]. We used the file gerp_conservation_scores.equus_caballus.EquCab3.0.bw obtained from the Ensembl FTP site (Release 113, [23]). The file with the GERP conservation scores was converted to a BedGraph format using the tool bigWigToBedGraph from the UCSC Genome Browser Utilities [24]. Subsequently, the genomic coordinates of the SNPs were aligned with the conservation scores using RStudio (version 2024.04.2, Posit Software, PBC, Boston, United States) to extract the GERP values for each SNP. This process enabled the identification of SNPs with high evolutionary conservation, allowing variants with potential functional relevance to be further prioritized.

#### 2.2.6. SNPs with Orthologous Locations in Regulatory Features of Human and Mouse Genomes

To determine whether the orthologous positions of SNPs (both markers and SNPs in LD) in mouse (*Mus musculus*) and human (*Homo sapiens*) genomes overlap with regulatory features, we used a combination of tools and datasets. First, the SNP coordinates were converted to BED format using the Python script Csvtobed. The liftOver tool [24] and the chain files equCab3ToHg38.over.chain and equCab3ToMm10.over.chain were used to map SNP coordinates from the EquCab3.0 horse reference genome to the GRCh38 (human) and GRCm39 (mouse) reference genomes. Regulatory feature files for human (homo_sapiens.GRCh38.Regulatory_Build.regulatory_features.20240230.gff) and mouse (mus_musculus.GRCm39.Regulatory_Build.regulatory_features.20240230.gff) were downloaded from the Ensembl FTP website (version 113, [23]). Finally, RStudio (version 2024.04.2, Posit Software, PBC, Boston, United States) was used to analyze the overlap between orthologous SNP positions and regulatory features in both species. This pipeline ensured the identification of SNPs located in regions of potential regulatory significance.

#### 2.2.7. SNPs with Potential Effect on Transcription Factor Binding

In this subsection, we identified SNPs that may affect transcription factor binding. SNP IDs with predicted effects were obtained from the agReg-SNPdb (Agriculture Regulatory SNP Database, https://azifi.tz.agrar.uni-goettingen.de/agreg-snpdb/, accessed 22 November 2024, [27]), which catalogs SNPs and their potential effects on transcription factor (TF) binding in promoter regions. The analysis considered the orientation of the gene to ensure accurate mapping of promoter regions to transcription start sites. This approach allowed us to identify SNPs that could affect regulatory mechanisms by altering TF binding sites, potentially impacting gene expression and associated traits.

#### 2.2.8. SNPs Within CpG Islands

To determine whether SNPs (both markers and SNPs in LD) are located within CpG islands, we used the file cpgIslandExt.txt.gz downloaded from the UCSC Genome Browser database (http://hgdownload.soe.ucsc.edu/goldenPath/equCab3/database/, accessed 26 November 2024). The file was converted to BED format and analyzed with RStudio (version 2024.04.2, Posit Software, PBC, Boston, United States) to identify overlaps between genomic SNP coordinates and CpG islands. This approach ensured the identification of SNPs located in these CpG-rich regions, which are often associated with gene regulatory functions such as methylation and transcriptional regulation.

#### 2.2.9. SNPs in 3′ UTR Regions Within Potential miRNA Binding Sites

The identification of SNPs in 3′UTR regions with potential miRNA binding sites was performed according to the procedure described in [28], using FASTA files from miRBase Release 22.1 (http://www.mirbase.org, [29]). Only mature miRNAs were considered, and putative target sites were defined as 6 nucleotides-long sequences in the genome representing the reverse complement of nucleotides 2 to 7 of the mature miRNA sequence. The genomic sequences were extracted with a window of ±5 bp around the SNP position, considering the gene orientation. Alignment and analysis were performed using RStudio (version 2024.04.2, Posit Software, PBC, Boston, United States). SNPs that can disrupt or create miRNA binding sites were identified, highlighting their potential regulatory significance in 3′ UTR regions.

#### 2.2.10. SNPs with Potential Effect on Alternative Polyadenylation

To determine whether SNPs could affect alternative polyadenylation, we used polyadenylation clustering data from the Animal-APAdb database (accessed 26 November 2024, [30]). Considering the gene orientation, an interval of 50 bp was added upstream or downstream of each polyadenylation cluster to capture potential regulatory regions. This window size was selected based on previous studies indicating that polyadenylation signals and regulatory motifs are typically located within 50 bp of cleavage sites, making this interval appropriate for capturing relevant SNPs [31,32]. RStudio (version 2024.04.2, Posit Software, PBC, Boston, United States) was used to compare the genomic coordinates of SNPs with these extended regions to identify overlaps. Using this approach, we were able to identify SNPs that may disrupt or create polyadenylation sites, potentially affecting gene expression and post-transcriptional regulation.

### 2.3. Cross-Species QTL Mapping for Meat Quality Traits

To identify the most promising candidate SNPs for horsemeat quality traits, we analyzed QTL data from other animal species. QTLs associated with meat quality traits were downloaded from the Animal QTLdb for cattle, chicken, goat, swine, and sheep (Release 54, accessed 19 November 2024, [33]). The 5000 bp was added upstream and downstream of each QTL coordinate to expand the region. Gene IDs within these expanded regions were retrieved using the Ensembl BioMart (Release 113, [16]). Subsequently, orthologous genes were identified in horses using g:Profiler (accessed 27 November 2024, [34]).

### 2.4. Functional Annotation and Pathway Analysis of Candidate Genes

To assess the biological significance of the identified genes, the STRING database (version 12.0, accessed 22 November 2024, [35]) was used to explore potential protein–protein interactions. Gene Ontology (GO) enrichment analysis for biological processes, cellular compartments, and molecular functions, as well as Kyoto Encyclopedia of Genes and Genomes (KEGG) pathway analysis, were performed using ShinyGO 0.81 [36]. In addition, parental KEGG orthology terms were extracted from the KEGG database (Release 112, accessed 4 December 2024, [37]) to better understand the functional roles of these genes. Cytoscape (version 3.10.1) was used for visualization [38].

## 3. Results

**Figure 1:**
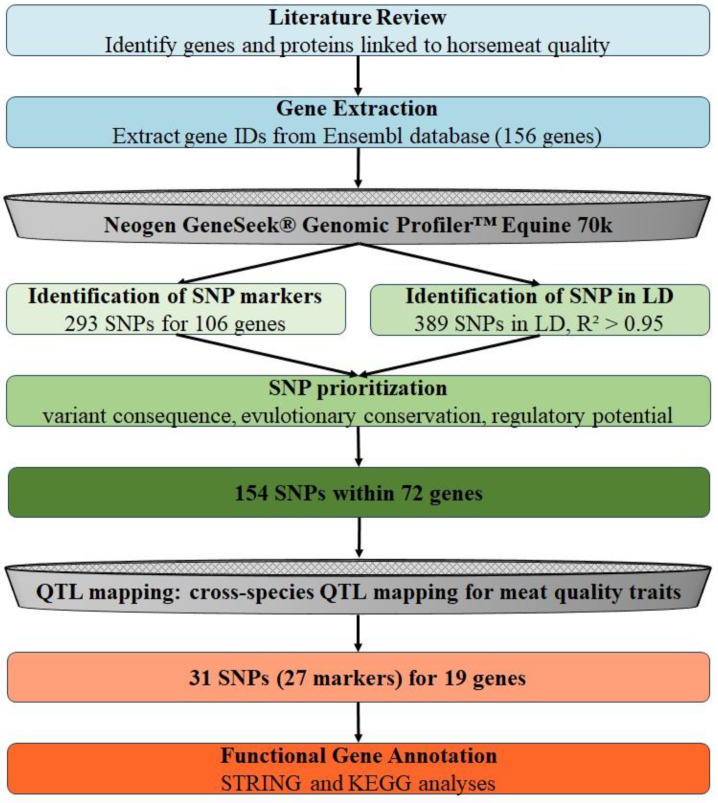
Workflow for identifying and prioritizing SNP markers for horsemeat quality traits.

This study uses a bioinformatics approach to identify and prioritize SNP markers for horsemeat quality traits. A systematic literature search was performed in PubMed to identify genes and proteins associated with meat quality traits in horses. SNP markers from the GeneSeek^®^ Genomic Profiler™ Equine Chip and SNPs in linkage disequilibrium (LD, R^2^ > 0.95) from the Animal-SNPAtlas database were included in the analysis. A total of 682 SNPs corresponding to 106 genes were initially evaluated based on predicted variant consequences, GERP scores, constrained element locations, orthologous regulatory regions in mice and humans, and potential effects on polyadenylation, miRNA binding, and transcription factor binding. Through this filtering process, the list was narrowed down to 154 SNPs associated with 72 genes. The final prioritization involved cross-species QTL mapping using QTL data from Animal QTLdb for cattle, chicken, goat, pig, and sheep. In horses, orthologous genes were identified, resulting in 31 SNPs corresponding to 27 SNP chip markers within 19 genes. These prioritized SNPs show their potential to improve horsemeat quality and support selective breeding programs. The workflow of the study is shown in Figure 1.

### 3.1. Literature Screening for Differentially Expressed Genes and Proteins

The comprehensive literature screening led to the successful identification of 156 genes, each of which was annotated with the Ensembl IDs for standardization and further analysis. These genes are potentially associated with the quality of horsemeat and influence traits such as tenderness, color, and flavor.

**Figure 2:**
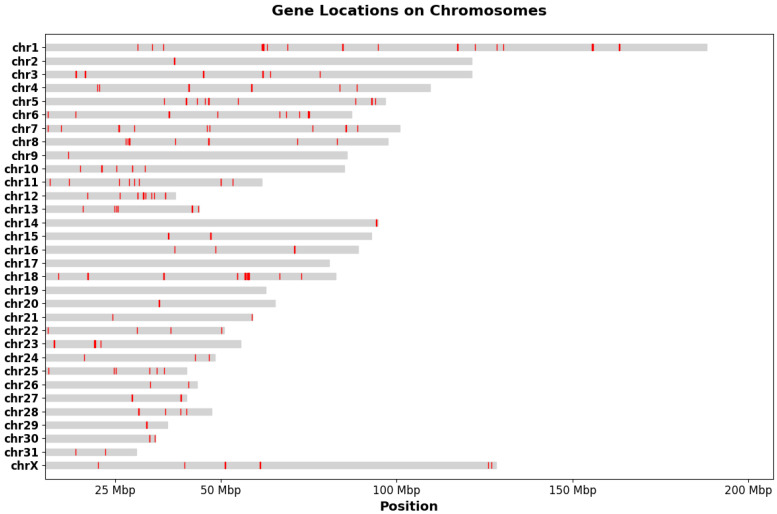
Gene distribution across horse chromosomes obtained from literature associated with horsemeat quality.

The distribution of these genes across chromosomes reveals an uneven pattern (Figure 2). Chromosome 1 hosts the highest number of genes (17 genes) and includes five distinct clusters, such as a prominent cluster spanning 61.63 Mbp to 68.96 Mbp with 5 genes. Chromosome 6 contains three clusters, including one spanning 66.63 Mbp to 75.09 Mbp with 5 genes, while chromosome 12 features the densest single cluster, with 11 genes spanning 11.98 Mbp to 34.27 Mbp. In contrast, chromosomes 17 and 19 lack any mapped genes in this dataset.

The analysis of the protein–protein interaction (PPI) network showed that 151 of the 156 identified genes were successfully imported into STRING, indicating a high representation of the dataset in the network. This indicates a strong functional and interactional relationship between most of the genes associated with horsemeat quality (Supplementary Figure S1).

The PPI network exhibited a high degree of connectivity, comprising 151 nodes and 801 edges, which significantly exceeded the expected number of edges (171), as confirmed by a PPI enrichment *p*-value of <1.0 × 10^−16^. The average node degree was 11.4, and the network had an average local clustering coefficient of 0.547, indicating a moderate level of local interconnectivity and interaction hubs within the network.

The network could be roughly divided into two major clusters: one comprising genes involved in metabolic processes important for energy production and biochemical processes, and another related to muscle cell development, differentiation, contraction, muscle structural development, myofibril assembly, and cytoskeletal organization. These clusters emphasized distinct but interrelated functional roles, with metabolic processes providing the biochemical basis for muscle function and structural development that directly impact meat quality.

**Figure 3:**
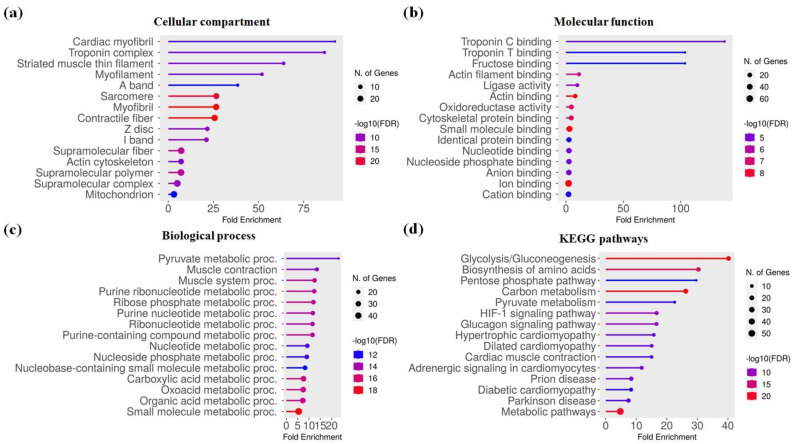
Gene Ontology (GO) enrichment analysis for (**a**) cellular compartments, (**b**) molecular functions, and (**c**) biological processes, as well as (**d**) Kyoto Encyclopedia of Genes and Genomes (KEGG) pathway analysis of genes associated with horsemeat quality. The analysis highlights significant involvement in muscle-specific components, metabolic processes, and key pathways such as glycolysis and amino acid biosynthesis.

In detail, the enrichment analysis showed that the identified genes were primarily associated with muscle-specific cellular components such as myofibrils, sarcomeres, and myofilaments, which are crucial for muscle structure and contraction. Molecular function analysis revealed significant enrichment in troponin C and T binding, actin filament binding, and cytoskeletal protein binding, indicating a role in muscle contraction and structural dynamics. The enrichment in fructose and oxidoreductase activity suggested links to metabolic processes. The biological process enrichment highlighted the involvement of these genes in muscle contraction, pyruvate metabolic processes, and small molecule metabolism, linking them to both muscle function and energy production. The analysis of KEGG metabolic pathways identified metabolic pathways such as glycolysis/gluconeogenesis, amino acid biosynthesis, and carbon metabolism, emphasizing the link of these genes to important metabolic and structural processes relevant to the horsemeat (Figure 3).

### 3.2. Identification and Expansion of SNP Markers from the GGP Equine Chip

Out of the 156 genes analyzed, we identified 293 unique SNPs, which were represented by 302 probes (markers) on the GGP Equine Chip, as some SNPs had multiple probes. These SNPs were associated with 106 genes, meaning that 50 genes did not have any corresponding markers on the chip. To expand the dataset and potentially include more functional SNPs, additional markers were identified using whole-genome sequencing data from the Animal-SNPAtlas database. SNPs in strong linkage disequilibrium (LD) with the chip markers (R^2^ > 0.95) were included, resulting in an additional 389 SNPs.

The analysis also identified SNPs in strong linkage disequilibrium (LD) within markers already present on the GGP Equine Chip. For instance, SNPs rs68820687 and rs68820688 in the gene *MYH2* exhibited an R^2^ of 0.955. Similarly, SNPs rs69127149 and rs69127154 in *NEB*, rs69126368 and rs69126372 in *MSTN*, rs69434178, rs69434180, and rs69434182 in *PRUNE2*, as well as rs69607214 and rs69607215 in *MSN*, all displayed perfect LD with an R^2^ of 1. These findings underscored the close relationships between certain markers within key genes on the chip, which may be relevant for traits linked to muscle function and horsemeat quality.

### 3.3. SNP Prioritization for Horsemeat Quality Traits

**Figure 4:**
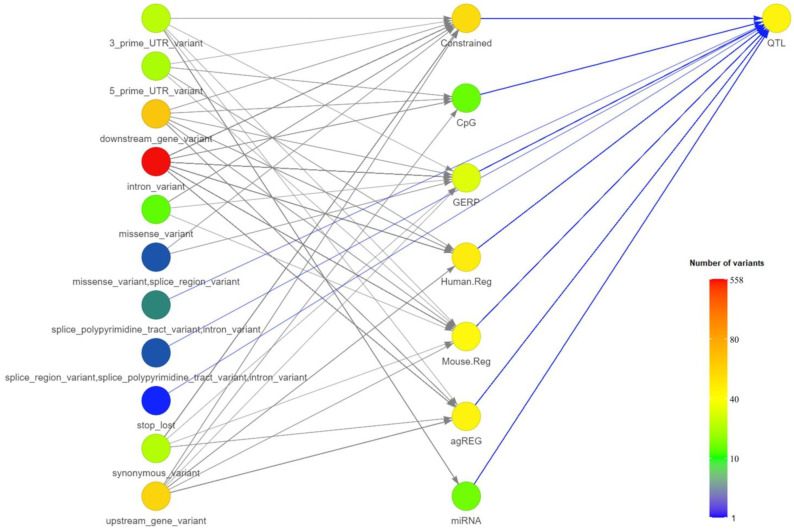
A network diagram showing the prioritization of SNPs based on various criteria. LEGEND: Constrained—variant within constrained element, CpG—variant within CpG island, GERP—variant with GERP score above 2, Human.Reg—orthologous location of the variants is in regulatory region in human, Mouse.Reg—orthologous location of the variants is in regulatory region in mouse, agREG—variant may affect transcription factor binding (according to agReg—SNPdb), miRNA—variant may affect miRNA binding.

SNPs were prioritized based on a comprehensive set of criteria, including variant consequence, GERP score, whether the SNP affects transcription factor binding, and its location, whether this is within a constrained element, an orthologous regulatory element in mouse or human, a miRNA seed binding region, near a polyadenylation cluster, or a CpG island. SNPs that met at least one of these criteria were considered for prioritization. The result was that 153 SNPs were associated with 72 genes (Supplementary Table S1). To further refine the list and identify stronger candidates, only SNPs within genes whose orthologs are in other species in QTLs associated with meat quality traits were considered. This additional filtering step resulted in a final selection of 31 SNPs in 19 genes that represent the most promising candidates for further investigation into horsemeat quality. The filtering process is visually summarized in Figure 4.

Of the 31 prioritized variants, 14 were directly identified as markers on the GGP Equine SNP Chip, while 17 were prioritized based on their linkage disequilibrium (LD) with WGS-identified SNPs, yielding a total of 27 markers for 19 genes. These variants were located in intronic regions, upstream and downstream gene regions, and UTR sequences, with some of them predicted to have missense effects (Table 1). Notably, several intronic variants overlapped constrained elements and regulatory regions, including rs69014980 in *ALDOA* (TFBS disruption) and *MACROD1* SNPs (orthologous enhancers). Variants in *ALB* and *PYGM* were predicted to alter TFBSs, while rs69611459 in *ASNS* and rs69522601 in *RAVER2* were in 3′ UTR regions overlapping with miRNA binding sites, suggesting potential post-transcriptional regulation. Among missense variants, SNPs in *AHNAK*, *MTHFD1*, and *PLIN1* may impact protein function, while a splicing variant in *RYR3* could affect transcript stability. A detailed breakdown of these variants, their functional annotations, and potential regulatory roles is provided in Table 1.

Although the final priority list focuses on the most promising candidates, it is important to note that several other potentially significant variants have been identified but have not been considered at this stage. For example, missense variants with high GERP scores, such as rs782900202 (6.29) in *BPGM* and rs69127149 (4.27) in NEB, are associated with traits such as muscle fibers and tenderness. Similarly, rs3435577028 in MYH2 (marker GGP_033_Risk_Factor_Immune_Mediated_Myositis_MYH1) and the stop-loss variant rs1150019112 in PGK1 remain strong candidates due to their associations with intramuscular fat (IMF), muscle type, and tenderness traits. Although they were excluded from the final prioritization, these variants merit further investigation.

### 3.4. Functional Annotation of Genes with Prioritized SNPs

The 31 prioritized SNPs are in 19 genes, many of which are associated with biological processes critical to muscle biology and meat quality. To understand their functional significance, these genes were analyzed for their association with meat quality traits such as tenderness and muscle structure based on evidence reported in the literature. In addition, their positions within protein–protein interaction (PPI) networks and KEGG pathway analyses were investigated to uncover their role in metabolic and cellular processes.

**Figure 5:**
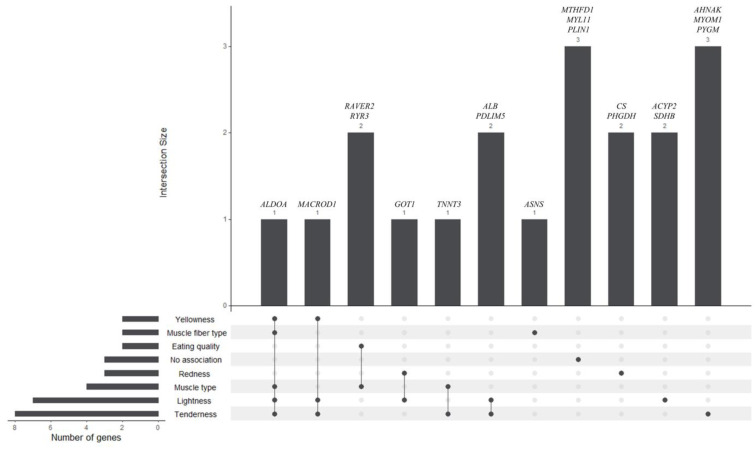
Associations of genes carrying prioritized SNPs with meat quality traits based on the literature data. The bar heights (Intersection Size) indicate the number of genes associated with the specific trait(s) shown below each bar. Dots and connecting lines represent shared associations between traits, while horizontal bars on the left display the total number of genes linked to each individual trait.

The literature-based associations, shown in Figure 5, highlight *ALDOA* and *MACROD1* as central genes, with *ALDOA* associated with five traits (tenderness, muscle fiber type, muscle type, lightness, and yellowness) and *MACROD1* associated with three traits (tenderness, lightness, and yellowness). These results emphasize their broad functional significance for traits affecting meat quality. In addition, *MYOM1* and *TNNT3* are associated with tenderness and muscle type, while *RAVER2* and *RYR3* show links to eating quality and muscle type, suggesting a role in muscle development and meat texture. Other genes, such as *GOT1* and *ALB*, are associated with redness and lightness, respectively.

**Figure 6:**
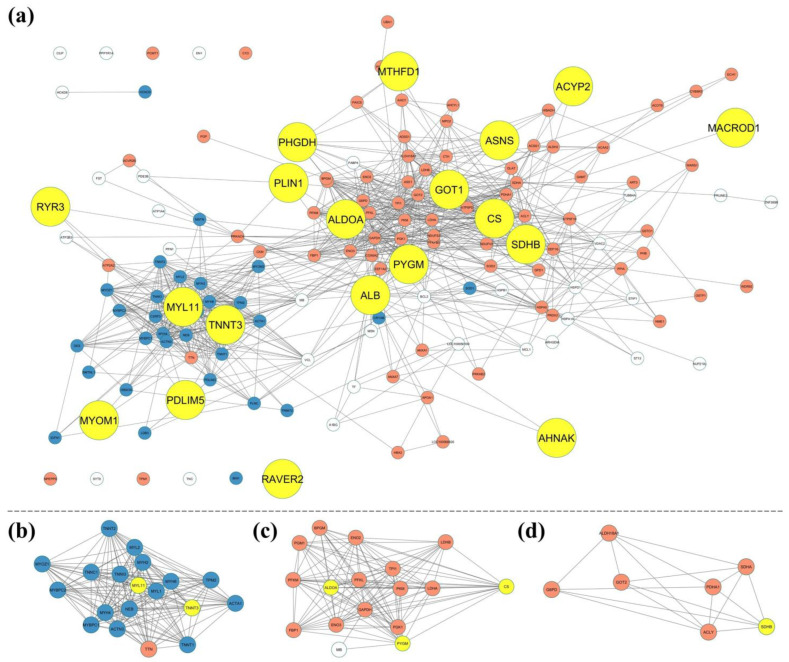
Protein–protein interaction (PPI) network showing the positions of the 19 prioritized genes (yellow nodes) within a broader network of interacting proteins. The red nodes represent proteins involved in metabolic processes, while the blue nodes correspond to proteins associated with muscle structure, development, and contraction. (**a**) Full PPI network illustrating interactions among the prioritized genes and other genes. (**b**–**d**) MCODE clustering was applied to the full PPI network (**a**), resulting in three functional clusters: (**b**) muscle structure cluster, highlighting genes related to muscle contraction and sarcomere organization, including *MYL11* and *TNNT3*, (**c**) metabolic processes cluster, featuring *ALDOA*, CS, and *PYGM*, and (**d**) mitochondrial energy metabolism cluster, including *SDHB*.

To investigate the functional interactions of the prioritized genes, they were mapped in a protein–protein interaction (PPI) network (Figure 6). Several prioritized genes, including *ALDOA*, *CS*, *PYGM*, and *SDHB*, occupy central positions within the network and interact closely with metabolic genes. These central hub genes emphasize their importance in maintaining biochemical balance in muscle tissue. In addition, genes such as *MYL11* and *TNNT3* are closely associated with structural genes related to muscle development, contraction, and myofibril assembly, underscoring their role in maintaining muscle structure and function, which are critical for traits such as tenderness and muscle type. Notably, *MACROD1*, *ACYP2*, *MTHFD1*, and *AHNAK* are found at more peripheral positions within the network, suggesting possible specialized roles or indirect interactions within these functional pathways. Similarly, *RYR3* is associated with both structural and metabolic groups, reflecting its functional versatility.

**Figure 7:**
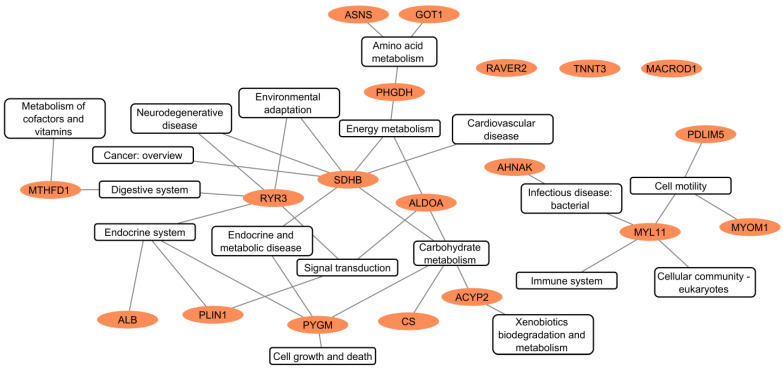
KEGG pathway analysis of 19 candidate genes for horsemeat quality analyzed using the KEGG database. Nodes circled in red indicate candidate genes with prioritized markers.

A further analysis of the KEGG pathways, summarized in Figure 7 and the Supplementary Table S2, illustrates the biological processes in which these genes are involved. Genes such as *ALDOA*, *CS*, and *SDHB* play a key role in carbohydrate metabolism and energy production, including the glycolysis, TCA, and oxidative phosphorylation pathways. *PYGM* is linked to several metabolic pathways, including carbohydrate metabolism (starch and sucrose metabolism) and the endocrine system (insulin and glucagon signaling pathways).

In addition to energy metabolism, several genes are also involved in amino acid metabolism. *ASNS* and *GOT1* are involved in alanine, aspartate, and glutamate metabolism, while *PHGDH* contributes to the metabolism of glycine, serine, threonine, cysteine, and methionine.

Genes associated with muscle structure and cytoskeletal organization include *MYL11*, *MYOM1*, and *PDLIM5*, which are involved in pathways related to focal adhesion, the regulation of the actin cytoskeleton, and cell motility. *RYR3* and PLIN1 are involved in apelin signaling and related pathways. The former is also involved in the digestive system. The same is true for *MTHFD1*, via the transport and metabolism of folate. *ALB* is involved in the synthesis of thyroid hormones. These associations reflect their broader biological functions, which may affect muscle development, energy balance, post-mortem metabolic processes, and ultimately meat quality.

## 4. Discussion

In this study, we focused on the identification of SNP markers on the GGP Equine SNP chip, which contains over 70,000 markers. A literature screening identified 156 genes associated with meat quality traits, aging, muscle fiber type, and muscle type, mainly involved in metabolism and muscle structure. From the chip, we obtained 302 markers for 293 SNPs associated with 106 genes. To expand the dataset and potentially include more functional SNPs, additional markers were first identified using whole-genome sequencing data from the Animal-SNPAtlas database. SNPs showing strong linkage disequilibrium (LD) with the chip markers (R^2^ > 0.95) were included in the dataset, resulting in an additional 389 SNPs. All SNPs were then analyzed for the predicted variant consequences, GERP scores, and positions in constrained elements and orthologous regulatory regions in the human and mouse genomes, as well as their effects on TF binding, alternative polyadenylation, and miRNA binding sites. This comprehensive approach resulted in a dataset of 153 SNPs associated with 72 genes. Through further filtering, only SNPs within genes whose orthologs are linked to QTLs for meat quality traits in other species were selected. This resulted in a final selection of 31 SNPs (27 SNP markers on the SNP chip) in 19 genes involved in metabolism, including lipid and amino acid pathways, muscle structure, signaling pathways, and regulatory functions, representing important candidates for further investigation.

### 4.1. Genes Involved in Energy Production and Metabolism

Energy production and metabolic pathways are critical for maintaining muscle function and have a direct influence on meat quality traits such as tenderness, intramuscular fat (IMF), and color. This section focuses on genes involved in glycolysis, the citric acid cycle (TCA), oxidative phosphorylation, and pyruvate metabolism. These processes collectively affect energy balance during muscle development and post-mortem aging, making these genes important targets for understanding and improving meat quality. Key genes in this category include *ALDOA*, *CS*, *ACYP2*, *SDHB*, and *PYGM*, each of which contributes uniquely to muscle energy dynamics and its influence on meat traits.

ALDOA (aldolase A) is essential and central to glycolysis and energy production in muscle cells, making it a critical player in meat quality traits such as tenderness, juiciness, and color parameters such as lightness (L*) and yellowness (b*) [10,15,39]. Among the prioritized SNPs, rs69014980 and rs782859809 were identified as intron variants. Both SNPs are in constrained elements, which emphasizes their evolutionary significance. In particular, rs782859809 leads to loss of TFBS, possibly altering gene regulation. Variations in *ALDOA* expression affect proteolysis of structural proteins during post-mortem aging, impacting tenderness and overall texture [15,40]. These results position *ALDOA* and its polymorphism rs782859809 as potentially valuable biomarkers and genetic targets for the improvement of horsemeat quality traits.

CS (citrate synthase) is crucial for the citric acid cycle (TCA), which is of central importance for mitochondrial energy metabolism. In horses, higher CS activity correlates with oxidative (type I) muscle fibers, which improves meat tenderness. Breed-specific differences, such as in Warmbloods and Quarter Horses, emphasize the role of CS in mitochondrial function and muscle composition, which influence traits of horsemeat such as IMF, tenderness, and pH [41]. In a study by López-Pedrouso et al., CS was associated with redness [15]. From the PPI network analysis in the present study, CS is part of a tightly linked cluster identified by MCODE within metabolic pathways, underscoring its central role in energy metabolism and its important influence on muscle. Among the prioritized SNPs, rs1143598220 is an intron variant. This SNP is located within an orthologous enhancer, suggesting possible regulatory effects on *CS* gene expression.

ACYP2 (acylphosphatase 2) plays a crucial role in carbohydrate/pyruvate metabolism. In a study by López-Pedrouso et al., it was associated with color parameters of horsemeat [15], which could be due to its influence on muscle fiber composition, as shown in mice [42]. Carroll et al. mapped quantitative trait loci (QTL) affecting differences in soleus muscle fiber properties between the LG/J and SM/J mouse strains. The LG/J strain, which had higher *Acyp2* expression, showed a greater proportion of type I oxidative muscle fibers and a larger cross-sectional area of type I and IIA fibers compared to SM/J [42]. These characteristics are associated with increased myoglobin content and enhanced oxidative metabolism, factors known to contribute to deeper red hues and the improved color stability of meat. In contrast to many of the candidate genes identified in this study, which are primarily involved in glycolytic metabolic pathways, ACYP2 is more aligned with oxidative metabolism. This distinction is reflected in its peripheral positioning within the PPI network and is supported by the KEGG enrichment analysis. Among the prioritized SNPs, rs397216526 and rs69086544 were identified as intron variants located within orthologous enhancers, suggesting a possible regulatory role for *ACYP2* expression.

SDHB (succinate dehydrogenase complex subunit B) is a key component of mitochondrial energy metabolism and influences traits such as tenderness, IMF content, and the overall quality of horsemeat. Its role in oxidative phosphorylation and muscle function underlines its importance for meat quality. Recent studies [15,39,43] have shown correlations between SDHB abundance and improved meat tenderness, which is supported by results in pigs in which SDH subunits affect growth and sensory traits [44]. The prioritized variant rs68545902 (in LD with BIEC2_471516), an upstream and intron variant, shows a score change in transcription factor binding, suggesting regulatory effects on *SDHB* expression and possibly affecting SDHB-driven signaling pathways associated with meat quality traits such as tenderness and IMF.

The *PYGM* gene, which encodes muscle glycogen phosphorylase, plays a crucial role in the breakdown of glycogen in muscle tissue. In horses, PYGM has been associated with variations in muscle composition and metabolism, traits that significantly influence meat production. This enzymatic activity is vital for muscle metabolism and has a direct impact on meat quality traits such as tenderness, color, and palatability. In horses, PYGM has been linked to variations in muscle composition and metabolism, traits that significantly influence meat production characteristics [45]. Among the prioritized variants, rs68895977 is an upstream gene variant. This variant is associated with a gain of TFBS, suggesting a possible regulatory role in *PYGM* expression. Research in cattle has shown that variants in *PYGM* can lead to conditions such as myophosphorylase deficiency, resulting in undesirable meat qualities such as high pH and dark cut beef—a condition that is undesirable for consumers and economically costly for producers [45]. In addition, the same variant rs68895977 has also been associated with body weight and size in Kazakh Zhabe horses, highlighting its importance for production traits [46]

### 4.2. Genes Involved in Protein and Amino Acid Metabolism

Amino acid and protein metabolism is crucial for muscle growth and for meat quality traits such as tenderness, marbling, and flavor. This section highlights *GOT1*, *ASNS*, and *PHGDH*, which regulate key processes such as amino acid biosynthesis, protein synthesis, and oxidative stress management and are therefore important candidates for improving meat quality.

GOT1 (glutamic-oxaloacetic transaminase 1) is an essential component of amino acid metabolism and plays a decisive role in influencing meat quality traits. In horses, its activity in energy metabolism and in the regulation of oxidative stress has been associated with variations in meat color profiles [15]. In addition, amino acid metabolism promoted by GOT1 contributes to the development of IMF, a key determinant of meat tenderness, marbling, and flavor. The increased expression of *GOT1* has been associated with enhanced lipid synthesis and adipocyte differentiation in pigs, both of which are critical for IMF accumulation [47]. This makes GOT1 an important factor for consumer-preferred meat traits such as improved flavor and texture. Among the prioritized SNPs, rs68640585 was identified as an upstream gene variant, 5′ UTR variant, and intron variant. This SNP overlaps with an orthologous enhancer that may have a regulatory influence on *GOT1*. These results emphasize that *GOT1* is a promising candidate gene for further study.

ASNS (asparagine synthetase), which is associated with muscle fiber type [13], is crucial for amino acid metabolism, especially for the conversion of aspartate and glutamine to asparagine [48]. Its activity is crucial for protein synthesis and muscle growth, processes that directly affect meat quality traits such as tenderness and flavor. The higher expression of *ASNS* has been associated with improved muscle development and stress resilience, highlighting its importance in livestock production [49]. Among the prioritized SNPs, rs69611459 is a 3′ UTR variant. This SNP is predicted to interact with miR-329b, which may be of functional significance given the reported differential expression of miR-329b in the muscles of Dorset and Small Tail Han sheep [50]. In addition, miR-329b is involved in pre-adipocyte differentiation [51], suggesting a potential role in influencing IMF content, which is an important determinant of meat quality. These results suggest that rs69611459 may be an important factor in improving the quality characteristics of horsemeat through its involvement in miRNA-mediated regulation of *ASNS*.

PHGDH (phosphoglycerate dehydrogenase) is essential for serine biosynthesis [52], a critical metabolic process that affects muscle development and metabolism [53]. López-Pedrouso et al. found a correlation between PHGDH abundance and horsemeat redness [15]. The higher abundance of PHGDH could positively regulate muscle cell proliferation, as demonstrated in chickens [54]. Interestingly, in pigs, the addition of 0.15% serine to the diet of finishing pigs promoted meat quality, which was characterized by higher IMF content and less drip loss [55]. Among the prioritized SNPs, rs394992807 and rs395235076 (both in LD with BIEC2_908679) were identified as intron variants located within orthologous enhancers, suggesting possible regulatory effects on *PHGDH* expression.

### 4.3. Genes Involved in Lipid Metabolism and Fat Deposition

Lipid metabolism and fat deposition are decisive factors for meat quality and influence traits such as tenderness, marbling, and flavor. Genes in this category regulate processes such as fat storage, fatty acid transport, and lipid biosynthesis. This section looks at *PLIN1*, *ALB*, and *MTHFD1* and highlights their role in intramuscular fat (IMF) storage, oxidative stability, and regulatory pathways that make them valuable candidates for improving horsemeat quality.

PLIN1 (perilipin 1) is a key regulator of lipid metabolism and fat deposition that influences meat quality in horses and other livestock. Studies in pigs [56] and chickens [57] have shown that *PLIN1* polymorphisms are associated with IMF content and fat deposition, which are important determinants of meat quality. Higher expression levels of *PLIN1* in adipose tissue have been associated with increased lipid accumulation, which affects both carcass traits and meat tenderness [56,57]. These results suggest that *PLIN1* is a promising candidate gene for genetic improvement strategies targeting meat quality. Among the prioritized SNPs, rs397479732 represents a missense variant that overlaps with an orthologous enhancer and possibly affects the regulatory function of *PLIN1* and its contribution to lipid metabolism. The further investigation of this variant could provide deeper insights into its role in equine meat production.

The *ALB* gene, which codes for serum albumin, plays a crucial role in lipid metabolism and fatty acid transport. Its functions in the mobilization of fatty acids and antioxidant activity in plasma influence muscle composition, oxidative stability, tenderness, and color [15]. Among the prioritized SNPs, rs68643854, located as an intron variant within a constrained element, shows evolutionary conservation, underscoring its potential functional significance. Similarly, rs68643900, an upstream gene variant that is in linkage disequilibrium with BIEC2_785750, is associated with a gain of TFBS. This suggests a possible involvement in regulatory functions that may influence *ALB* expression and thus meat quality. These results position *ALB* and its polymorphisms as a candidate gene for further investigation into its contribution to horsemeat quality and genetic improvement programs.

*MTHFD1* (methylenetetrahydrofolate dehydrogenase, cyclohydrolase, and formyltetrahydrofolate synthetase 1) encodes a trifunctional enzyme associated with choline/folate-dependent one-carbon metabolism. In donkeys, it has been associated with IMF [58], which is not surprising since folate (essential B vitamin) intake and folate status have been linked to changes in the expression of genes involved in lipid metabolism [59]. In particular, the expression of *MTHFD1* is lower in the longissimus dorsi muscle of Wagyu cattle compared to Chinese Red Steppe cattle [60], and a human missense variant rs2236225 was associated with increased body mass index [61]. Among the prioritized SNPs, rs782854427, a missense variant in LD with BIEC2_632813 and within a constrained element, potentially alters the function of the encoded protein, highlighting its evolutionary and functional significance. In addition, the intronic variant rs69302674, which is also within a constrained element, suggests regulatory significance. These results position *MTHFD1* and its polymorphisms as promising candidates for further investigation into their role in improving the quality traits of horsemeat.

### 4.4. Genes Involved in Muscle Structure and Function

Muscle structure and function are of central importance for meat quality. Genes in this category regulate muscle contraction, the stability of sarcomeres, and the composition of fiber types. These processes have a direct effect on traits such as tenderness, texture, and IMF content. This section highlights *TNNT3*, *MYOM1*, *MYL11*, *PDLIM5*, *AHNAK*, and *RYR3*, which play a central role in muscle dynamics and post-mortem processes and are therefore important targets for the genetic improvement of meat quality.

TNNT3 (troponin T3) is an important component of the troponin complex that regulates muscle contraction in fast skeletal muscle fibers. The composition and distribution of muscle fiber types has a significant influence on meat quality, such as tenderness. Fast-twitch fibers, which express TNNT3 particularly strongly, are often associated with variations in tenderness due to their different metabolic activity and structural properties during post-mortem aging [39,62]. The enzymatic degradation of muscle proteins after slaughter facilitates myofibril fragmentation, a process that positively correlates with improved tenderness. TNNT3 plays an important role in this process by influencing the stability and degradation of myofibril proteins, making it a promising target for breeding programs aimed at improving meat quality traits [39,40,63]. These results position *TNNT3* as a valuable gene for genetic improvement strategies in horse production systems. Among the prioritized SNPs, rs68882259 was identified as a downstream gene variant. Despite its downstream position, this SNP overlaps with an orthologous enhancer, potentially enabling distal enhancers to regulate *TNNT3* expression and subsequently influence meat quality traits, particularly tenderness.

MYOM1 (encoding myomesin-1) is a structural protein that is crucial for the stability of sarcomeres and muscle function. Increased levels of MYOM1 have been associated with improved tenderness, suggesting its potential as a biomarker for meat quality [15,64]. Among the prioritized variants, four intron variants—rs1142180487, rs1150182996, rs396225196, and rs68759950—were identified in linkage disequilibrium with the markers BIEC2_1047070, BIEC2_1047100, and BIEC2_1047096. The variant rs1142180487, located within a constrained element, emphasizes its evolutionary significance, while rs396225196 and rs68759950 are associated with orthologous enhancer regions, suggesting possible regulatory functions. The rs1150182996, which is linked to BIEC2_1047100, stands out due to its enhancer association and a high GERP score of 2.04, indicating significant regulatory potential.

The *MYL11* gene, which codes for myosin light chain 11, plays a crucial role in muscle contraction and structural organization and thus influences the quality traits of horsemeat. This gene may contribute to the balance between slow-twitch and fast-twitch muscle fibers, with a higher proportion of slow-twitch fibers being associated with better tenderness and flavor [65]. Although the specific role of MYL11 in horsemeat quality remains poorly understood, its essential function in muscle dynamics and energy metabolism suggests its potential influence on meat traits. Proteomic studies have linked MYL11 to important muscle-related signaling pathways that influence meat quality parameters such as texture and tenderness [39]. Among the prioritized SNPs, rs69065907 is an upstream gene variant associated with TFBS modifications, including loss, score changes, and gain of TFBS. These could affect the expression of *MYL11* and thereby impact muscle composition and associated meat quality traits. Further functional studies on this variant could provide deeper insights into its role in improving meat production traits in horses.

PDLIM5 (PDZ and LIM domain 5), a cytoskeleton-associated protein, influences muscle growth, differentiation and post-mortem processes, and its expression is associated with muscle fiber composition and meat tenderness [39,66]. Proteomic studies [15,39] have identified PDLIM5 as a marker for foal meat quality, highlighting its role in traits such as tenderness and IMF content. Among the prioritized SNPs, rs68623490 is an intron variant with a high GERP score of 4.91, located within a constrained element, indicating its evolutionary conservation and potential regulatory significance. The further investigation of this variant could improve our understanding of its functional effects and support its use in breeding programs to improve horsemeat quality.

Proteomic studies suggest that the AHNAK (AHNAK nucleoprotein), a protein involved in cytoarchitecture, influences post-mortem meat aging and tenderness through its association with stress-related annexin genes that promote meat tenderness [39,67]. In the present study, we prioritized a missense variant rs69004899 with potential functional significance.

RYR3 (ryanodine receptor 3) plays a central role in calcium signaling, a process that is essential for muscle function and development. As a subtype of the ryanodine receptor family, RYR3 regulates the release of calcium from the endoplasmic reticulum and thus directly influences muscle excitability, contraction, and fiber type conversion [68]. This gene plays a role in the conversion of fast-twitch (type II) fibers to slow-twitch (type I) fibers, which are finer and associated with improved oxidative metabolism, tenderness, and IMF. These properties significantly improve meat quality. The upregulation of *RYR3* in mares has been associated with improved muscle fiber characteristics in Kazakh horses, suggesting its importance for muscle-specific gene expression and fiber type differentiation [14]. In pigs, the *RYR1* gene polymorphism, a homolog of RYR3, has a significant effect on meat quality, with the CC genotype being associated with superior meat characteristics compared to CT [69]. The recessive homozygote is associated with pale, soft, exudative (PSE) meat [70]. These results emphasize the importance of ryanodine receptors in influencing traits such as tenderness and oxidative metabolism in different animal species. Among the prioritized variants, rs68480423 stands out as a splice polypyrimidine tract variant that may influence splicing efficiency and transcript stability. In addition, two SNPs (rs1142172760 and rs1148992878) in LD with rs1148992878 (BIEC2_65875) overlap with orthologous enhancers, suggesting a regulatory role of these SNPs. These results emphasize the functional significance of *RYR3* in modulating muscle composition and its potential as a genetic marker for improving quality traits in horsemeat.

### 4.5. Genes Involved in Regulatory Functions and Cellular Processes

RAVER2 (ribonucleoprotein, PTB binding 2), which is involved in RNA binding and alternative splicing, plays a crucial role in muscle-specific gene expression and fiber type differentiation. In a study by Wang et al. in Kazakh horses, RAVER2 was found to be upregulated in mares, highlighting its involvement in type I muscle fibers, which are finer and correlate with better meat quality compared to type II fibers [14]. In addition to horses, *RAVER2* has been identified as a candidate for positive selection in multi-omics analyses of Jining Gray and Boer goats and has been associated with traits such as pH and tenderness in cattle and sheep [71]. These results suggest its potential as a biomarker for meat quality across livestock species. Among the prioritized SNPs, rs69522601 is a synonymous and 3′ UTR variant of *RAVER2* that overlaps with conserved miRNA binding sites, including miR-509a-5p, miR-217, and miR-8924. Notably, miR-509a-5p is highly expressed in mature (24-month-old) skeletal muscle of donkeys compared to young (2-month-old) muscle, indicating its role in muscle development and maturation [72]. Therefore, rs69522601 may affect the post-transcriptional regulation of *RAVER2*, underscoring its functional importance in muscle composition and meat quality.

Recent proteomic analyses have identified MACROD1 (mono-ADP ribosylhydrolase 1) as a potential biomarker for monitoring meat quality traits. In a study by López-Pedrouso et al., the Burguete and Jaca Navarra horse breeds and their feeding regimes were compared. MACROD1 was found to affect the tenderness and lightness (L*) of the Burguete horse [15]. Among the prioritized SNPs, rs68893372, rs68893379, and rs69007205 are intron variants located within orthologous enhancers. Notably, rs69007205 is located within a constrained element, highlighting its evolutionary conservation and potential functional significance.

## 5. Conclusions

This study identified important SNP markers in genes involved in metabolism, muscle structure, signaling pathways, and fat metabolism, which may influence key horsemeat quality traits, such as tenderness, marbling, and color. Notable candidate genes include *ALDOA*, *CS*, *PYGM*, *PLIN1*, and *SDHB* (metabolic pathways) and *MYL11*, *TNNT3*, *MYOM1*, *PDLIM5*, and *RYR3* (muscle structure and function), which play a critical role in energy production, muscle composition, and post-mortem meat quality dynamics. These results provide valuable genetic insights for improving horsemeat quality through targeted breeding strategies. However, as we relied on bioinformatics-based selection and strict inclusion criteria, some relevant markers may have been excluded, and the lack of experimental validation limits their immediate applicability. To increase the reliability of these findings, future research should focus on integrating these markers into larger genomic studies, such as GWAS or GBLUP, to assess their association with meat quality traits and refine their predictive power. Additionally, functional validation is needed to confirm their biological effects. Ultimately, the inclusion of validated markers in genomic selection models will enhance breeding precision and contribute to advancements in equine meat quality research.

## Figures and Tables

**Table 1:** Prioritized SNPs in genes associated with horsemeat quality traits.

Gene Symbol	Type ^1^	RS ID	Marker ID ^2^	Variant Consequence	Constrained Element	GERP Score	TF Binding	Human Reg. ^3^	Mouse Reg. ^3^	miRNA Binding
*ACYP2*	LD	rs397216526	(BIEC2_307086)	intron variant		−0.446		enhancer		
*ACYP2*	LD	rs69086544	(BIEC2_307086)	intron variant		−0.446		enhancer		
*AHNAK*	Marker	rs69004899	12-26376598-G-A	missense variant		-				
*ALB*	Marker	rs68643854	BIEC2_825788	intron variant	Yes	−0.108				
*ALB*	LD	rs68643900	(BIEC2_785750)	upstream gene variant		−3.12	Gain of TFBS			
*ALDOA*	Marker	rs69014980	BIEC2_215098	intron variant	Yes	−0.27				
*ALDOA*	Marker	rs782859809	13-20698094-G-C	intron variant		3.87	Loss of TFBS			
*ASNS*	Marker	rs69611459	BIEC2-860417	3′ UTR variant		-				miR-329b
*CS*	LD	rs1143598220	(BIEC2_1184909)	intron variant		-			enhancer	
*GOT1*	Marker	rs68640585	BIEC2_13411	upstream gene variant, 5′ UTR variant, intron variant		−1.05			enhancer	
*MACROD1*	Marker	rs68893372	BIEC2_195029	intron variant		−5.12		enhancer	enhancer	
*MACROD1*	Marker	rs68893379	BIEC2_195036	intron variant		-			enhancer	
*MACROD1*	Marker	rs69007205	BIEC2_195018	intron variant	Yes	−0.807			enhancer	
*MTHFD1*	Marker	rs69302674	BIEC2_632816	intron variant	Yes	−0.303				
*MTHFD1*	LD	rs782854427	(BIEC2_632813)	missense variant	Yes	−1.43				
*MYL11*	Marker	rs69065907	BIEC2_214942	upstream gene variant		−1.72	Loss of TFBS, Score Change, Gain of TFBS			
*MYOM1*	LD	rs1142180487	(BIEC2_1047070)	intron variant	Yes	0.206				
*MYOM1*	LD	rs1150182996	(BIEC2_1047100)	intron variant		2.04		enhancer		
*MYOM1*	LD	rs396225196	(BIEC2_1047096)	intron variant		0.623		enhancer		
*MYOM1*	Marker	rs68759950	BIEC2_1047096	intron variant		−0.836			enhancer	
*PDLIM5*	Marker	rs68623490	BIEC2_818877	intron variant	Yes	4.91				
*PHGDH*	LD	rs394992807	(BIEC2_908679)	intron variant		-			enhancer	
*PHGDH*	LD	rs395235076	(BIEC2_908679)	intron variant		-			enhancer	
*PLIN1*	Marker	rs397479732	CUHSNP00113433	missense variant		−0.63			enhancer	
*PYGM*	Marker	rs68895977	BIEC2_195234	upstream gene variant		0.644	Gain of TFBS			
*RAVER2*	Marker	rs69522601	BIEC2_978964	synonymous variant, 3′ UTR variant	Yes	−4.36				miR-217, miR-509a-5p, miR-8924, miR-1388, miR-9065, miR-9124
*RYR3*	LD	rs1142172760	(BIEC2_65875)	intron variant		−8.78			enhancer	
*RYR3*	LD	rs1148992878	(BIEC2_65875)	intron variant		-			enhancer	
*RYR3*	Marker	rs68480423	BIEC2_66277	splice polypyrimidine tract variant, intron variant		−5.06				
*SDHB*	LD	rs68545902	BIEC2_471516	upstream gene variant, intron variant		−1.79	Score Change			
*TNNT3*	Marker	rs68882259	BIEC2_200114	downstream gene variant		-		enhancer		

^1^ Indicated whether the prioritized SNP is directly represented as a marker (Marker) on the SNP chip or is in linkage disequilibrium (LD) with a marker on the SNP chip. ^2^ Markers in brackets indicate SNP markers on the SNP chip that are in LD with the prioritized SNP. ^3^ Indicated the orthologous location of prioritized marker is within regulatory element in human (Human.Reg) and mouse (Mouse.Reg).

## Data Availability

The original contributions presented in this study are included in the article/Supplementary Material. Further inquiries can be directed to the corresponding author.

## Supplementary Materials

The following supporting information can be downloaded at https://www.mdpi.com/article/10.3390/foods14030533/s1: Figure S1: Protein–protein interaction (PPI) network of 151 genes associated with horsemeat quality; Table S1: List of 153 prioritized SNPs associated with 72 genes; Table S2: KEGG pathway annotations for prioritized genes, classified into hierarchical levels: KEGG Level 1 (broad functional category), KEGG Level 2 (metabolic or functional subcategory), and KEGG Level 3 (specific pathway).
