# Supplementary figures and images for: Exploring Genetic Influences on Equine Meat Quality: A Bioinformatics Approach
Source: Foods. 2025 Feb 6;14(3):533. doi: 10.3390/foods14030533 (PMC11816967; doi:10.3390/foods14030533)

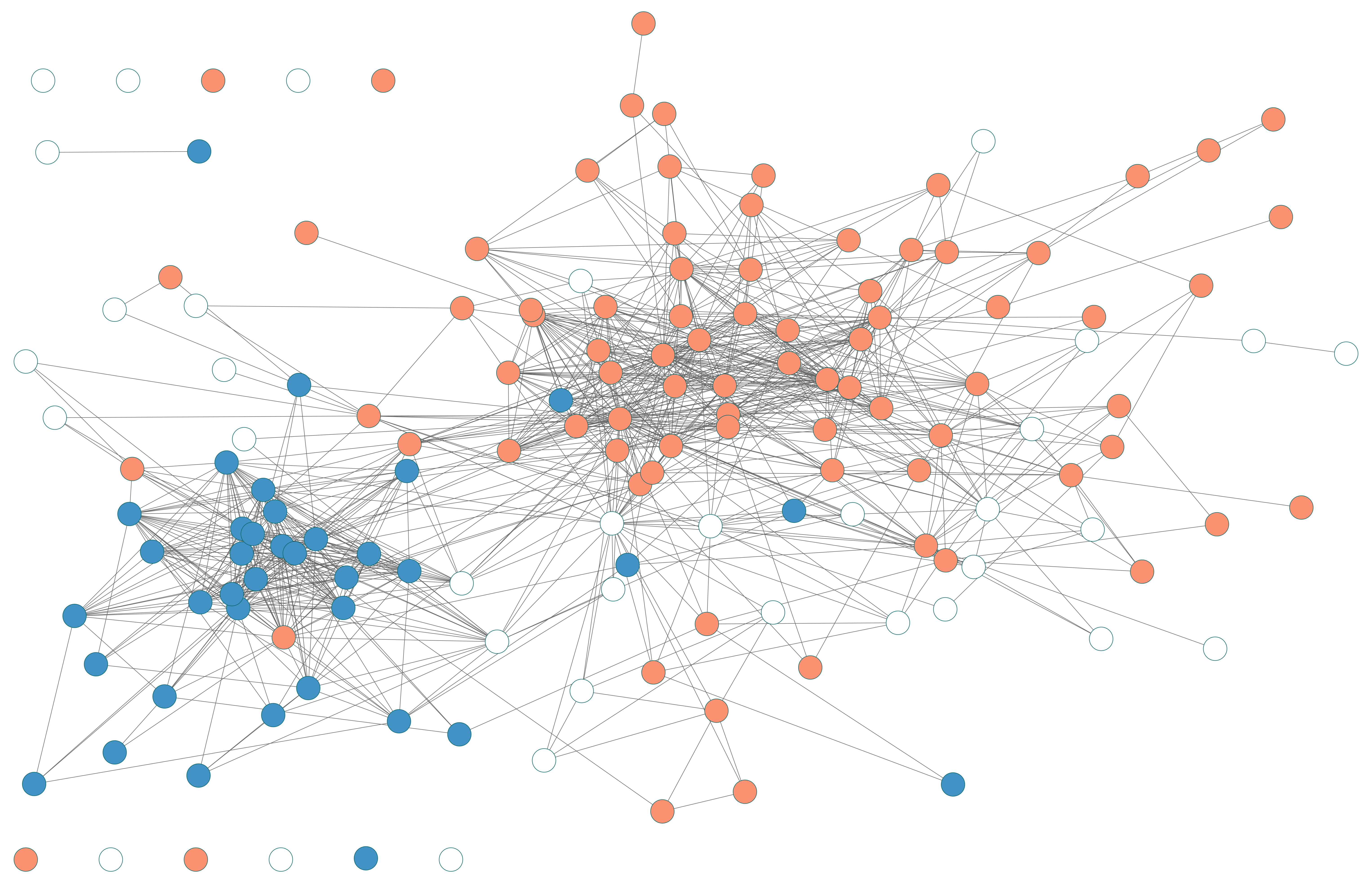

Supplement: Supplementary file 1 [file foods-14-00533-s001.zip › Figure S1.png]
